# Liposomal Entrapment or Chemical Modification of Relaxin2 for Prolongation of Its Stability and Biological Activity

**DOI:** 10.3390/biom12101362

**Published:** 2022-09-24

**Authors:** George Kogkos, Foteini Gkartziou, Spyridon Mourtas, Kostas K. Barlos, Pavlos Klepetsanis, Kleomenis Barlos, Sophia G. Antimisiaris

**Affiliations:** 1 Lab Pharm Technology, Department of Pharmacy, University of Patras, Rio, 26504 Patras, Greece; 2 Foundation for Research and Technology Hellas, Institute of Chemical Engineering, FORTH/ICE-HT, Platani, 26504 Patras, Greece; 3 Department of Chemistry, University of Patras, Rio, 26504 Patras, Greece; 4 Chemical & Biopharmaceutical Laboratories CBL Patras, Ind. Area of Patras, Block 1, 25018 Patras, Greece

**Keywords:** peptides, relaxin, lipidic conjugate, liposomes, stability, biological activity

## Abstract

Relaxin (RLX) is a protein that is structurally similar to insulin and has interesting biological activities. As with all proteins, preservation of RLX’s structural integrity/biological functionality is problematic. Herein, we investigated two methods for increasing the duration of relaxin-2’s (RLX2) biological activity: synthesis of a palmitoyl RLX2 conjugate (P-RLX2) with the use of a Palmitoyl-l-Glu-OtBu peptide modifier, and encapsulation into liposomes of P-RLX2, RLX2, and its oxidized form (O-RLX2). For liposomal encapsulation thin-film hydration and DRV methods were applied, and different lipid compositions were tested for optimized protein loading. RLX2 and O-RLX2 were quantified by HPLC. The capability of the peptides/conjugate to stimulate transfected cells to produce cyclic adenosine monophosphate (cAMP) was used as a measure of their biological activity. The stability and bioactivity of free and liposomal RLX2 types were monitored for a 30 d period, in buffer (in some cases) and bovine serum (80%) at 37 °C. The results showed that liposome encapsulation substantially increased the RLX2 integrity in buffer; PEGylated liposomes demonstrated a higher protection. Liposome encapsulation also increased the stability of RLX2 and O-RLX2 in serum. Considering the peptide’s biological activity, cAMP production of RLX2 was higher than that of the oxidized form and the P-RLX2 conjugate (which demonstrated a similar activity to O-RLX2 when measured in buffer, but lower when measured in the presence of serum proteins), while liposome encapsulation resulted in a slight decrease of bioactivity initially, but prolonged the peptide bioactivity during incubation in serum. It was concluded that liposome encapsulation of RLX2 and synthetic modification to P-RLX2 can both prolong RLX2 peptide in vitro stability; however, the applied chemical conjugation results in a significant loss of bioactivity (cAMP production), whereas the effect of liposome entrapment on RLX2 activity was significantly lower.

## 1. Introduction

Relaxin 2 (RLX2) is a two-chain peptide hormone that has a similar structure to insulin. It is one of the peptides of the so called “insulin-relaxin peptide family” that includes insulin, insulin-like growth factors, relaxins (1, 2, and 3), and relaxin/insulin-like factors (3, 4, 5, and 6) [1]. RLX1, RXL2, and RLX3 differ in their origins and amino-acid sequence [1]. RLX2 is currently considered a pleiotropic hormone, and it has important functions in the brain, heart, and kidney, in addition to its role in nitric oxide regulation and neo-angiogenesis [1,2]. RLX2′s therapeutic potential has recently been connected with its ability to prevent the tissue remodeling observed in fibrosis and to conserve endogenous tissue structure [3], while it has been demonstrated to contribute to bone remodeling/expansion [4]. Furthermore, recombinant human relaxin 2 is currently being clinically evaluated for treating acute heart failure [5].

Chemically, RLX2 consists of two peptide chains, the A-chain and the B-chain, that are linked by two cysteine bridges [6,7]. The chemical synthesis of RLX2 was not achieved until recently, due to the low solubility of the B-chain, and the low yield achieved when combining the A and B chains. The solubility of RLX2 B-chain was markedly increased by oxidation of its Met^25^, and thus effective solid-phase synthesis was recently achieved [8]. Chemical synthesis of RLX2 facilitates its use as a therapeutic; however, the intrinsic limitations of peptides have impeded the development of peptide drugs, including RLX2. The two major constraints on peptide drug development are their poor oral bioavailability and short half-life in the bloodstream, due to serum enzyme susceptibility and rapid renal clearance [9]. In particular, RLX2 has a very short in vivo half-life of approximately 10 min in rabbits and monkeys [10], and also in women [11]. For this reason, it was necessary to administer RLX2 continuously for 48 h by intravenous infusion into patients, in clinical studies (Phase III trials) that investigated the capability of RLX2 as a therapeutic for treating acute heart failure [5]. Therefore, there is a clear need for improving the pharmacokinetic properties of RLX2, in order to potentially improve its therapeutic value. Numerous chemical approaches to address this concern have been undertaken in recent years. Conjugating the peptide to an inert chemical moiety is one of the strategies repeatedly proven to be successful in extending the half-life of various peptide therapeutics [9]. More specifically, a meta-analysis of studies that involved fatty acid conjugates of peptides indicated that acylation contributed to a statistically significant extension of the half-life of the peptides [12]. The replacement of disulfide bonds with non-reducible elements has been demonstrated to be effective and to eliminate the relaxin-specific deleterious effect of serum reductases. In particular, substitution with dicarba bonds via ring closure metathesis has been increasingly applied to many bioactive cystine-rich peptides [13].

An insulin dimer was recently demonstrated to be thermodynamically stable in vitro [14]. It was thus proposed that perhaps the dimeric form of RLX2 may also be more stable, compared to its monomeric form. However, although dicarba peptide analogues of RLX2 were found to retain strong receptor activity, their stability in serum was drastically reduced [13]. Nevertheless, other synthetic covalently linked dimeric forms of RLX2 were seen to retain their receptor binding activity and additionally demonstrated improved in vitro serum stability [15].

Another possibility for the enhancement and prolongation of peptide stability is to encapsulate them into nanometer sized vesicles, as a method to protect them from denaturation (proteolysis and dilution effects). For this reason, several encapsulation methodologies have been optimized to achieve a high encapsulation of chemically unstable molecules, but in the end they could not preserve their functionality [16,17]. Elsewhere, it was reported that the liposome preparation method that denatures the least protein is the lipid film hydration technique, but a drawback of this method is that the encapsulation efficiency achieved is low [18,19]. Previously, using a modified film hydration method, it was demonstrated that 40% of initial acetylcholinesterase (AChE) could be encapsulated in a functional state in liposomes, but the encapsulated enzyme had reduced activity, due to reduced substrate permeation through the lipid membrane. When the permeability of the liposome membrane was modified by a porin, full function of the enzyme was recovered [20].

Herein, we evaluated and compared both methods discussed above, for the first time, for their ability to protect RLX2 peptides from degradation and loss of biological activity, in vitro. In particular, we synthesized a novel palmitoyl conjugate of RLX2 peptide (P-RLX2), using the Palmitoyl-l-Glu-OtBu peptide modifier. This peptide modifier is known to effectively extend the peptide shelf-life of Liraglutide (Victoza), by delaying its absorption and reducing its renal clearance, due to the shielding effect of the fatty acid moiety [21]. Additionally, we developed novel types of liposomes that encapsulate RLX2, as well as its oxidized form (O-RLX2) and the P-RLX2 conjugate. For liposome formation, the dehydration-rehydration vesicle (DRV) method was applied; a mild method that protects the integrity of peptide/protein drugs and confers high encapsulation for aqueous soluble materials, as reported in [22,23].

## 2. Materials and Methods

### 2.1. Materials

Egg phosphatidylocholine (PC), 1,2-Distearoyl-*sn*-glycerol-3-phosphatidyl ethanolamine *N*-[methoxy (polyethylene-glycol)-2000] (PEG), and Phosphatidyl-glycerine (PG) were purchased from Lipoid, Germany. Cholesterol (Chol) was purchased from Sigma-Aldrich (Darmstadt, Germany). Human Relaxin 2 (RLX2) and Oxidized Human Relaxin 2 (O-RLX2), as well as *N*-Palmitoyl-l-glutamic acid α-tert-butyl ester γ-succinimidyl ester (Palmitoyl-l-Glu(OSu)-OtBu), were kindly supplied by CBL (Patras, Greece). Agar was from Sigma-Aldrich (Darmstadt, Germany). All solvents used were of analytical or HPLC grade and purchased from Merck (Darmstadt, Germany). All other materials, such as the salts used for buffer preparation, reagents for lipid concentration determination, etc., were of analytical grade and were purchased from Sigma Aldrich (Darmstadt, Germany).

### 2.2. Characterization (HLPC, ESI-MS)

HPLC analysis was performed on a Shimadzu LC-2010 liquid chromatography system (Canby, OR, USA) using a LiChrospher^®^ 100 RP-18 (5 μm) LiChroCART^®^ 250-4. Mobile phase: THF/H_2_O; Gradient: 20% THF to 100% THF in 30 min (Conditions A); 50% THF to 100% THF in 30 min (Conditions B); Flow rate: 1 mL/min; Detection at 280 nm. ESI-MS spectra were recorded on a Waters Micromass ZQ 4000 mass detector (positive mode), controlled by MassLynx 4.1 software, by direct infusion, using a syringe pump at a flow rate of 5 μL/min. Cone voltage was set at 30 V and scan time at 1 s, with the interscan delay at 0.1 s.

### 2.3. Synthesis and Analysis of N^α^-(Palmitoyl-l-γ-Glutamyl-α-OtBu)-RLX2 (P-RLX2)

RLX2 (50 mg; 8.39 μmol) was dissolved in buffer phosphate PB at pH 7.40 (2 mL). To the resulting solution, Palmitoyl-l-Glu(OSu)-OtBu (18.08 mg; 4 eq) dissolved in DMSO or Dioxane (2 mL) was added dropwise at 4–5 °C while vigorously stirring, within 2 h, and the reaction mixture was further stirred for 12 h at 20 °C. Next, the reaction mixture was dialyzed over distilled water (3 times) and then lyophilized to afford a white powder that was further purified by semi-preparative HPLC. The collected fractions of P-RLX2 were combined and finally lyophilized to afford P-RLX2 as a white solid (yield 30–35% relative to RLX2). Purity: > 98% (determined by hplc analysis). ESI-MS [M+3H] calcd.: 2128.38; found: 2129.46; [M+4H] calcd.: 1596.54; found: 1597.26; [M+5H] calcd.: 1277.43; found: 1278.05; [M+6H] calcd.: 1064.69; found: 1065.28.

### 2.4. Preparation of RLX2 Peptide Liposomes

The lipid membrane compositions used for all the liposome formulations prepared were (i) PC/Chol (2:1 mole/mole), (ii) PC/PG/Chol (8:2:5 mole/mole/mole), and (iii) PC/PG/Chol/PEG (8:2:5:1.3 mole/mole/mole/mole). Details about liposome preparation using the two different methods applied are mentioned below.

#### 2.4.1. Thin-Film Hydration Method

The thin film hydration method was used for P-RLX2 liposome preparation, as previously described in detail [24]. In brief, the lipids required for lipid composition of PC/PG/Chol/PEG (8:2:5:1.3, mol/mol), together with the required amount of P-RLX2 (to give a P-RLX2/lipid molar ratio of 0.003, and a total lipid concentration of 10 mg/mL), were dissolved in a chloroform/methanol (2:1 *v*/*v*) mixture. The mixture was then placed in a round-bottom flask and connected to a rotor evaporator (BÜCHI Labortechnik AG, Postfach, Switzerland) under vacuum, until complete evaporation of the organic solvents and the formation of a thin lipidic film on the sides of the flask. The film was hydrated with 1 mL of PBS at 40 °C and vortexed until complete removal of the lipids from the sides of the flask, for multilamellar vesicle (MLV) formation.

#### 2.4.2. Dehydration-Rehydration Vesicle Method

For dehydration–rehydration vesicle (DRV) preparation, empty small unilamellar vesicles (SUV) were initially prepared as described in detail before, from empty MLV liposomes [22,23,24]. For this, the lipids were dissolved in a chloroform/methanol (2:1 *v*/*v*) mixture and stored at −20 °C. For each liposome preparation, the appropriate amounts of each solution (depending on the lipid composition required), for a final lipid concentration of 20 mg/mL, were placed in a 100-mL round-bottomed flask. Organic solvents were evaporated by the connection of the flask to a rotary evaporator, until a thin film was formed. For complete removal of organic solvents, the film was flashed-dried with nitrogen for 2–3 min. The lipid film was hydrated with 1 mL of diluted PBS buffer (10% *v*/*v*) pH 7.40, heated at 40 °C. The resulting dispersions consisting of MLVs were subsequently converted into SUVs by probe sonication with a tapered micro-tip (Vibra cell, Sonics and Materials, Suffolk, UK). In all cases, the initially turbid liposomal suspension was well clarified after sonication for 5–15 min. Following sonication, the liposome suspensions (SUV) were left to stand for at least 1 h at 40 °C, in order to anneal any structural defects. Any Ti-fragment and/lipid aggregate contaminants were removed from SUV suspensions by centrifugation at 15,000× *g* for 20 min. Then, 1 mL of the SUV suspension was mixed with 1 mL of a 0.5 mg/mL RLX2 or O-RLX2 solution (in distilled H_2_O), and the mixture was freeze-dried. With controlled rehydration of the dried materials, as described previously [22,23], multilamellar DRVs were generated.

#### 2.4.3. Liposome (MLV and DRV) Size Reduction and Purification

Size reductions of liposomes (both MLVs and DRVs) were carried out by sequential extrusion of the liposomal dispersions at least ten times, through polycarbonate filters with a pore diameter 0.4 µm, followed by a second extrusion cycle through membranes with a 0.1 µm pore diameter, fitted in a syringe-type extruder (Lipo-so-fast, Avestin, Ottawa, ON, Canada). Extrusion was used as a size-reduction method, since sonication was avoided, in order to prevent disruption of the DRV liposomes and leakage of the encapsulated drug and/or destruction of the encapsulated protein. After size reduction, all liposomes were purified from non-entrapped (or incorporated) peptides by size exclusion chromatography on a Sepharose CL-4B column (1 × 40 cm), eluted with PBS, pH 7.40.

#### 2.4.4. Post-Pegylation of Liposomes

For preparation of PEGylated liposomes, a post-pegylation technique [25] was used, since it was found that the protein encapsulation in DRV liposomes was low when PEG-lipid was added in the lipid phase during DRV preparation. For this, PEG-lipid micellar dispersion was prepared using the thin film hydration method at a final concentration of 11.88 mg/mL of PEG-lipid in PBS, followed by an annealing step at 40 °C for 30 min. Next, a PEG dispersion (11.88 mg/mL) and liposomal dispersion (20 mg/mL), prepared as previously described by the DRV method, were mixed at a volume ratio 1:1, *v*/*v* and the mixture was incubated at 40 °C for 2 h. After post-pegylation, liposomes were purified from non-incorporated PEG, by size exclusion chromatography, as described above.

### 2.5. Physicochemical Characterization of RLX2 Peptide Liposomes

#### 2.5.1. Encapsulation Efficiency (EE%)

Liposomes were characterized for peptide encapsulation efficiency (%), calculated according to Equation (1):

(1) $$\mathrm{EE}\;(\%)=\frac{\frac{D}{L}\left(final\right)\;\left(\frac{mol}{mol}\right)}{\frac{D}{L}\;\left(initial\right)\;\left(\frac{mol}{mol}\right)}\cdot 100$$

where D is drug (peptide) concentration and L is lipid concentration; initial means before purification and final after liposome purification. Liposome lipid concentration was measured using a Stewart assay [26], a colorimetric method used for the quantification of phospholipids.

RLX2 and O-RLX2 concentration in liposomes was quantified by gradient high-performance liquid chromatography (HPLC) using a Shimadzu 20A5 Gradient HPLC system coupled to a SPD-20A Prominence UV/VIS detector operating at 220 nm. A Luna^®^ 5 µm C18 (2) 100 Å, LC Column (250 × 4.6 mm) was used; the mobile phase was a mixture of acidified water (0.1% *v*/*v* trifluoroacetic acid) and acetonitrile at 63:37 *v*/*v*. The column was eluted at a flow rate of 1 mL/min at 25 °C, and RLX2 or O-RLX2 was eluted at 5.2 min and 5.4 min, respectively. The sample injection volume was 100 µL. A calibration curve in the range of 2.5–30 µg/mL was constructed by preparation of standard solutions of RLX2 or O-RLX2 in the presence of 1 mg/mL lipid (lipid composition PC/Chol or PC/PG/Chol) in media with a similar composition to the samples. Liposomes were analyzed after being lysed in isopropanol, for efficient protein extraction, as previously reported [27,28]. One volume of sample (150 μL) was mixed with one volume (150 μL) of isopropanol, and the mixture was agitated in a vortex, followed by the addition of 300 μL HEPES buffer (pH 5.0) at a stable final lipid concentration of 1 mg/mL. For the quantification of the peptides encapsulated in the liposomes of lipid composition PC/PG/Chol/PEG, a similar procedure, with a few modifications, was applied. In particular, a calibration curve was constructed in the range of 2.5–15 µg/mL, by preparation of standard solutions of RLX2 or O-RLX2 in the presence of 1 mg/mL lipid (lipid composition PC/PG/Chol/PEG) in media with similar composition as the samples in each case. Liposomes were analyzed after being lysed in isopropanol; for this, 100 μL of liposomal dispersion was mixed with 150 μL of isopropanol, and the mixture was agitated by a vortex, followed by the addition of 300 μL HEPES buffer (pH 5.0) at a stable final lipid concentration of 1 mg/mL.

Due to the low absorptivity of the conjugate P-RLX2, its concentration in the liposomes could not be measured by the HPLC method (as used for calculation of the yield of the synthetic procedure), and its complete incorporation in the liposomes was verified by HPLC studies carried out in the combined liposome and free molecule fractions collected, following size exclusion chromatography on a Sepharose CL-4B column (1 × 40 cm), eluted with PBS, pH 7.40.

#### 2.5.2. Vesicle Physicochemical Properties

The particle size distribution (mean hydrodynamic diameter and polydispersity index) of RLX2, O-RLX2, and P-RLX2 loaded liposomes dispersed at 0.4 mg/mL lipid, in phosphate-buffered saline (10 mM) with pH 7.40, was measured by dynamic light scattering (DLS) (Malvern Nano-Zs, Malvern Instruments, Malvern, Worcestershire, UK) at 25 °C and a 173° angle [24]. Each sample was measured 11 times in three independent measurements. The polydispersity index (PDI) was used as a measure of homogeneity of liposomal dispersions. Dispersions having a PDI of less than 0.200 or 0.250 are generally considered to have a narrow size distribution. Zeta potential was measured in the same dispersions, at 25 °C, utilizing the Doppler electrophoresis technique, as recently reported [24].

#### 2.5.3. RLX2 Peptide Stability Studies in Buffer and Serum (In Vitro)

To ensure peptide stability during incubation in media containing, or not containing, serum proteins, solutions of RLX2 peptides, as well as liposomal dispersions with lipid membrane compositions of PC/PG/Chol (8:2:5 mol/mol/mol) and PC/PG/Chol/PEG (8:2:5:1.3 mol/mol/mol/mol), were incubated at a final peptide concentration of 35 ± 8.1 μg/mL (when PBS was used as media), and 58 ± 5.0 μg/mL (when FBS (80% *v*/*v*) was used as media). Samples were placed separately in hermetically sealed screw-tubes (to avoid evaporation) and then in an orbital incubator (Stuart S1500, UK) set at 50 rpm and 37 °C, for a period of up to 30 d. At specific time intervals, samples were analyzed by HPLC, for quantification of intact RLX2 or O-RLX2, with the method mentioned above, in the case of samples in PBS. For quantification of free or liposomal peptides in the presence of 80% FBS, serum proteins precipitation was accomplished by mixing one volume of sample (100 μL) with three volumes (300 μL) of acidified isopropanol (with 0.1% TFA), followed by vortex agitation and centrifugation at 15000 rpm for 15 min. Next, the supernatant was transferred to a new eppendorf tube and 400 μL of HEPES (pH 5.0) were added, followed by vigorous vortex for 1 min, in order to retrieve the peptide. Finally, the samples were analyzed by HPLC, based on a calibration curve constructed at the exact same conditions as the samples to be analyzed.

For direct comparison of the stability of the different peptides, the results were normalized and expressed as the percentage of the corresponding initial peptide concentration measured at time point 0, for each peptide and each formulation (solution or liposome). Experiments were carried out in triplicate, and the results are expressed as the mean value ± standard deviation.

### 2.6. RLX2 Peptide Biological Activity Evaluation (In Vitro)

#### 2.6.1. Cell culture, Transfection, and Stimulation for cAMP Production

Human HEK-293 embryonic kidney cells (HEK) (American Type Culture Collection, Manassas, VA, USA) were provided by Prof. G.T. Stathopoulos (Medical School, University of Patras, Patras, Greece). Cells were grown in Dulbecco’s modified Eagle’s medium (D-MEM; Gibco BRL, Gaithersburg, MD, USA) supplemented with 10% fetal bovine serum (FBS; Biosera, Nuaille, France), 100 IU penicillin/mL, and 100 μg streptomycin/mL (Gibco, Gran Iskand, NY, USA), and incubated at 37 °C and 5% CO_2_. A total of 6 × 10^5^ cells were seeded in 6-well plates and allowed to attach overnight. The following day, cells were transected with RXFP1 (LGR7, OriGene, Rockville, MD, USA) with Xfect transfection reagent (Takara, San Jose, CA, USA). After 48 h, RXFP1-HEK293 cells with transient expression were prepared for stimulation assay, with various receptor agonists. In more detail, cells were washed twice with Hanks’ balanced salt solution and were incubated in Hanks’ balanced salt solution in the presence of 0.1 mM 3-isobutyl-1-methylxanthine (IBMX, stimulation buffer, Sigma-Merck, Darmstadt, Germany) for 10 min. Cells were then stimulated for 30 min with vehicle or RLX2 peptide types at concentrations between 0.1–100 nM, media were aspirated, and 200 μL of HCL 0.1 M was added into each well, to extract cAMP. After 10 min, the extracts were collected and centrifuged at 600 g for 5 min and subsequently samples were used for Cyclic adenosine monophosphate (cAMP) assay [29]. HEK293 cells without drug stimulation were employed as the control group.

#### 2.6.2. Cyclic Adenosine Monophosphate (cAMP) Assay

The biological activity of RLX2 peptides was measured by their ability to stimulate cAMP production in RXFP1-HEK293 cells [29]. The dose–response bioactivity of the three peptide types evaluated was measured at peptide concentrations 0.1, 1, 10, and 100 nM. cAMP concentration was measured according to the instructions of the cAMP Direct Immunoassay Kit (ab65355, Abcam, Cambridge, UK). For this, after carrying out the stimulation procedure as described above, and prior to quantification, cAMP standards and samples were neutralized and acetylated using the neutralizing buffer and acetylating reagent supplied in the kit, respectively. For quantification, 50 μL of standard cAMP solutions or 50 µL of samples were added to the Protein G coated 96-well plate provided. After blending with 10 μL cAMP antibody, the suspension was incubated for 1 h at room temperature with gentle agitation and for another 1 h after adding 10 mL of cAMP-HRP. Then the plate was washed 5 times, and the wells were incubated with 100 μL of HRP substrate for 1 h. The reaction was stopped using 100 μL of 1Μ HCl, and absorbance at 450 nm was measured using a microtiter plate reader (FLUOstar Omega, BMG LABTECH). The molar concentration of cAMP was determined from standard curves generated using standard solutions, normalized to the corresponding protein concentrations and measured using the Bradford Protein Assay (Thermo Scientific) and finally expressed as pmol cAMP/μg protein. Each experiment was performed in triplicate, and the results are expressed as mean ± S.D.

#### 2.6.3. Preservation of Bioactivity of Liposomal and Free RLX2 Peptides

For these experiments, RLX2 peptides (400 nM) in the form of solutions (free) or liposomes (with lipid membrane composition: PC/PG/Chol/PEG, 8.0:2.0:5.0:1.3 mol/mol) were incubated in FBS (80% *v*/*v*) at 37 °C, for 30 d. At specific time points (0, 1, 3, 4, 10, and 30 d), 250 μL of each sample was added to 750 μL of culture medium (4× dilution), in order to realize a peptide concentration of 100 nM for all tested samples. HEK-293 cells were transfected and stimulated with each compound, and the cAMP concentration was measured, following the procedures described above. Experiments were carried out in triplicate, and the results are expressed as mean value ± standard deviation.

### 2.7. Statistical Methods

The IBM SPSS statistics pack was used for the statistical analysis of the results. All experiments were performed in triplicate. All data are presented as the mean ± standard deviation of the mean of independent experiments. Statistical significance was evaluated using one-way ANOVA or two-way ANOVA and LSD’s post hoc test with a significance level of *p* < 0.05.

## 3. Results

### 3.1. RLX2 Peptide Quantification

**Figure 1:**
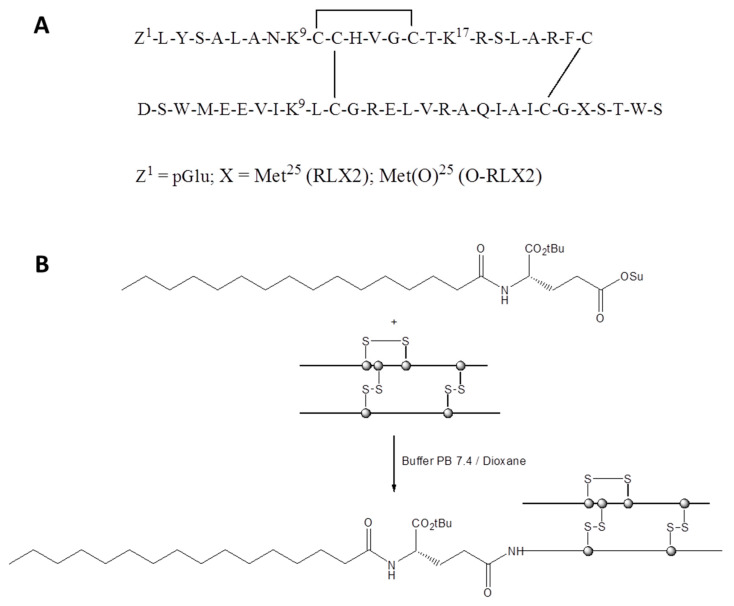
(**A**) Structure of human relaxin 2 (RLX2) and Met (O)^25^-human relaxin 2 (O-RLX2). (**B**) Synthesis of P-RLX2 by the reaction of Palmitoyl-l-Glu(OSu)-OtBu and human relaxin 2 (RLX2).

The structure of RLX2 and O-RLX2 peptides are presented in Figure 1A. All calibration curves constructed for the measurement of RLX2 and O-RLX2 peptides, in solution or in liposomes, as well as those that were constructed in additional presence of serum proteins (together with liposome or not) were linear in the concentration ranges evaluated, with linear correlation coefficient R^2^ values highest than 0.991 in all cases, as seen in Figures S1–S4 (supplementary data files). All samples were measured within the concentration range of the corresponding calibration curves, for each case, verifying the accuracy of the results.

### 3.2. Synthesis of N^α^-(Palmitoyl-l-γ-Glutamyl-α-OtBu)-RLX2 (P-RLX2)

For the synthesis of P-RLX2, we considered the site-selective reaction of *N*-Palmitoyl-l-glutamic acid α-tert-butyl ester γ-succinimidyl ester (Palmitoyl-l-Glu(OSu)-OtBu) at the a-amino group of the *N*-terminal amino acid of the Β-chain of RLX2, avoiding acylation at the ε-amino group of Lys9,17 of the A chain and the ε-amino group of Lys9 of the B chain. In addition, the use of pGlu at the *N*-terminal of the A-chain of RLX2 (Figure 1) proved that lipidation at the a-amino group of this amino acid is not feasible. Such regioselectivity would enable the synthesis of P-RLX2 by introducing palmitoyl fatty acid attached to the amino group of glutamic acid (which is used as a spacer), at the a-amino group of the *N*-terminal amino acid of the Β-chain of RLX2, by the side chain carboxylic group of Glu, at the last step of its synthesis, according to Figure 1B.

**Figure 2:**
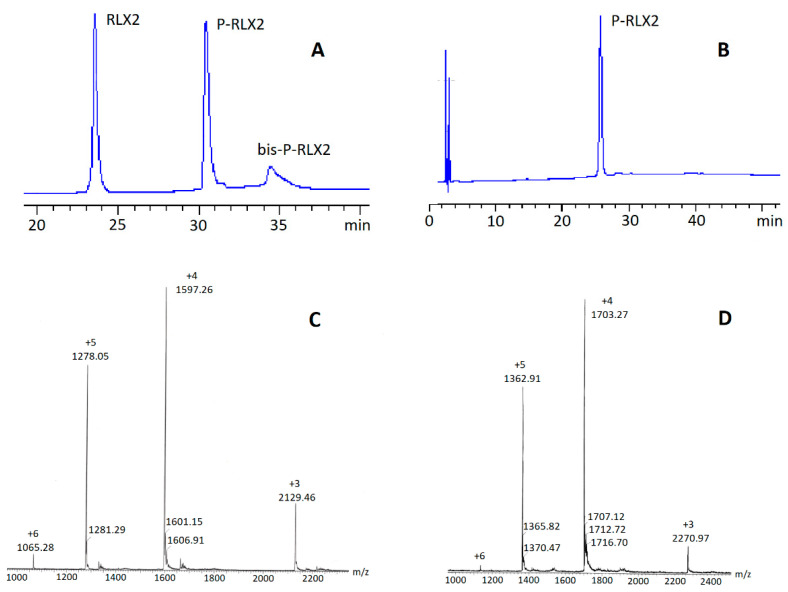
(**A**) Representative analytical HPLC of reaction mixture (conditions A). (**B**) Representative analytical HPLC of purified P-RLX2 (conditions B). (**C**) ESI-MS of P-RLX2. (**D**) ESI-MS of bis-P-RLX2.

In order to achieve selective lipidation at the *N*-terminus of the B-chain of RLX2, we considered dissolving RLX2 in phosphate buffer (PB) at pH 7.40. Thus, the reaction was performed in PB 7.40 or a mixture of PB 7.40 and DMSO (or Dioxane), by dropwise addition of up to a four molar excess of Palmitoyl-l-Glu(OSu)-OtBu to an aqueous solution of RLX2. The reaction progress was monitored by HPLC analysis. With this method, P-RLX2 was formed in a percentage of about 35–50% relative to RLX2, depending on the exact reaction conditions. Adding higher than a four molar excess of Palmitoyl-l-Glu(OSu)-OtBu repeatedly resulted in the formation of high amounts of bis-palmitate products (and possibly products with more palmitoyl groups being conjugated), as determined by a ESI-MS analysis of the corresponding eluted HPLC peaks. In Figure 2A a representative HPLC chromatograph is presented, where, besides the desired P-RLX2 (eluted at 30.5 min), the formation of bis-palmitate RLX2 (bis-P-RLX2) is also seen (eluted at 34.5 min). Both P-RLX2 and bis-P-RLX2 were identified by ESI-MS (Figure 2C,D, respectively). On the other hand, the use of up to a four molar excess of Palmitoyl-l-Glu(OSu)-OtBu resulted to the formation of P-RLX2, with only small amounts (and in some cases with no) of bis-palmitated RLX2. Then, the reaction mixture was subjected to dialysis over distilled water, and finally lyophilized and purified by semi-preparative HPLC, to afford, after lyophilization, P-RLX2 of > 98% purity. The overall yield, relative to RLX2, was about 30–35%. A representative analytical HPLC of the final obtained pure P-RLX2 conjugate that was used in the current studies is presented in Figure 2B.

### 3.3. Liposome Properties

The properties of the liposomal formulations of RLX2 are reported in Table 1. As seen, all the liposomes of RLX2 that were prepared by the DRV method are nanosize vesicles, with mean diameters between 113 and 159 nm, and the liposome dispersions have a narrow size distribution, judged by the low PDI values measured (0.0075–0.198) for all the formulations (different lipid compositions). Representative graphs of size distribution (by intensity) for all liposome types are shown in Figure S5 (supplementary data files).

It can be observed that the encapsulation of RLX2 in liposomes is markedly increased (from 4% to 22%), when the negatively charged lipid (PG) is added in the liposome membrane, due to the opposite charge attraction between the peptide (positively charged at this pH value) and the liposome membrane (that bears a negative charge, due to the presence of PG).

When PEG is added in the liposome membrane the EE of RLX2 drops to a very low value (down to 2.85%, from 22% when encapsulated in non-PEGylated liposomes). The fact that the zeta potential of the vesicles decreased when PEG was added proves that the coating of the liposome surface with PEG chains was achieved [30]. Nevertheless, the low RLX2 encapsulation suggests that the conventional method of preparing RLX2-loaded and PEGylated DRV liposomes could not be used. In another project, we recently reported that PEG addition in PC, and PC/Chol (2:1) liposomes prepared by the DRV method, resulted in a significant decrease of moxifloxacin encapsulation [31], and this was attributed to the “cryoprotectant-like” effect of PEG, which restricts complete disruption of empty SUV liposomes during the freeze-drying step of DRV formation, resulting in lower amounts of lipid membranes being available for re-structuring of liposomes and concurrent encapsulation of the drug during the hydration step [22,23].

For this reason, as mentioned in the Methods section (Section 2.4.4), in order to prepare PEGylated RLX2-loaded liposomes with high EE, non-PEGylated RLX2 loaded PC/PG/Chol DRV liposomes (selected due to their higher encapsulation efficiency) were initially prepared, and PEGylation was carried out in a second step. As seen in Table 1, incubation of the pre-formed RLX2-loaded PC/PG/Chol liposomes with PEG micelles for 1 h or 2 h periods did not make any difference in terms of the final amount of RLX2 peptide loaded into the PEGylated liposomes; however, the longer incubation period resulted in better coating with PEG, as judged from the greater decrease of the zeta-potential of the liposomes, when 2 h incubation was used (compared to 1 h). Although the PEGylated RLX2 liposomes prepared by this method had a slightly reduced EE%, compared to the non-PEGylated liposomes with the same lipid composition (approx. 17.4% to 22%), their EE was dramatically higher compared to the PEGylated liposomes prepared by the conventional methodology (2.85%). For this reason, the same post-PEGylation approach was carried out for formation of PEGylated O-RLX2 loaded liposomes.

In Table 2, the properties of O-RLX2 and P-RLX2 incorporating liposomes (the latter prepared by thin film hydration method) are reported. As seen, the size distribution (mean diameter between 138–154 nm, and PDI between 0.09–0.202) and surface charge of these liposomes are similar to the liposomes that encapsulate RLX2 (with corresponding lipid compositions) (Table 1).

Concerning the encapsulation efficiency, O-RLX2 was observed to obtain a similar encapsulation in liposomes to that of RLX2, implying that using the oxidized form of the peptide does not affect its encapsulation in liposomes (at least under the conditions applied herein). On the other hand, as a lipid conjugate, the Palm-L-Glu-OtBu conjugate of RLX2 (P-RLX2) is totally incorporated in the liposome lipid membrane (0.003 mole% (compared to total lipid)) using the thin film hydration method.

### 3.4. RLX2 Peptide Stability in Buffer and Serum-Effect of Liposome Encapsulation

**Figure 3:**
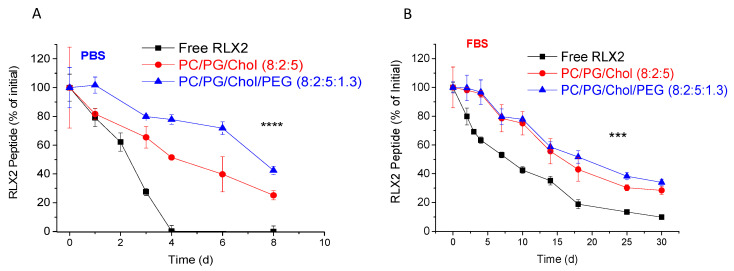
Stability of RLX2 peptide during incubation for 30 d at 37 °C of free or liposomal formulations in PBS (pH 7.40) (**A**), or in the presence of FBS (80% *v*/*v*) (**B**). All experiments were conducted at least 3 times, and the reported values are the mean values; bars represent the SD of each mean value. **** *p* < 0.0001; *** *p* = 0.0003.

In Figure 3, the stability of RLX2 during incubation in PBS (Figure 3A) and in FBS (Figure 3B) is presented. As seen, liposomal encapsulation significantly increased the peptide stability during incubation in both media tested. In fact, PEGylated liposomes provided longer protection of RLX2 during incubation in PBS buffer, while during incubation in FBS; both liposome types (non-PEGylated and PEGylated) had a similar effect on RLX2 stability. However, it is clearly demonstrated by comparing the results in Figure 3A,B, that the stability of the peptide was significantly higher, for longer time periods, when the peptide was incubated in the presence of proteins (FBS) compared to plain buffer; proteins thus seem to provide some protection against chemical degradation of the peptide in an aqueous environment. A similar effect was previously observed for curcumin [32], a polyphenolic substance that is rapidly degraded in aqueous environments, and this was attributed to the protection of curcumin from degradation by binding to serum proteins [33]. Nevertheless, we cannot be sure about the mechanism behind the increased stability of RLX2 peptide in the presence of proteins (compared to PBS); more experiments should be carried out to elucidate any potential shielding effect of the serum proteins towards RLX2 peptide, however this is outside of the scope of the current investigation. Furthermore, it should also be pointed out that the initial concentration of RLX2 peptide that was incubated in PBS was almost half of that incubated in FBS, so this difference could also be, at least partially, implicated in the different stabilities demonstrated in the two media.

In any case, regardless of the media in which the peptides were placed, encapsulation of RLX2 peptide into PEG liposomes resulted in a profound increase of its stability (Figure 3).

**Figure 4:**
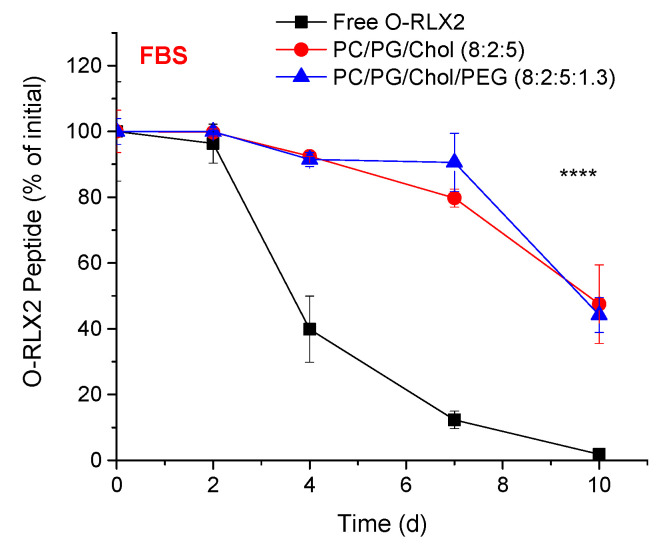
Stability of O-RLX2 peptide during incubation for 10 d at 37 °C of free or liposomal peptide in the presence of FBS (80% *v*/*v*). All experiments were conducted at least 3 times, and the reported values are the mean values; bars represent the SD of each mean value. **** *p* < 0.0001.

A similar stability study for O-RLX2 peptide in FBS showed that the oxidized form of the peptide (Figure 4) was significantly less stable compared to RLX2 peptide during incubation in FBS (Figure 3B). Indeed, although RLX2 peptide was observed to have a half-life of 7.54 d in FBS, under the same incubation conditions the oxidized form of the peptide had a half-life of 3.48 d. Thereby, these results indicate that the chemical modification applied in O-RLX2 (oxidation of Met^25^ B-chain), conferred a decrease of the peptide stability. Nevertheless, encapsulation in liposomes (PEGylated or not) provides prolonged stabilization of the oxidized form of RLX2; indeed, although after 10 d of incubation, all the free O-RLX2 peptide was degraded, liposomal encapsulation preserved more than 40% of the O-RLX2 peptide with the same incubation time period (Figure 4).

Interestingly, both types of RLX2 peptides were similarly protected when incubated in the presence of proteins by both PEGylated and non-PEGylated liposomes. The former observation was not expected, since it is well known that PEGylated liposomes have a higher integrity compared to non-PEGylated ones (in the presence of serum proteins, FBS) [24,30,31]. Perhaps the protection provided to the peptides that leaked out from the liposomes by the proteins in FBS compromised the different integrities of the two liposome types.

### 3.5. Biological Activity of RLX2 Peptide Types

#### 3.5.1. Initial Activity of the Various Peptide Types

**Figure 5:**
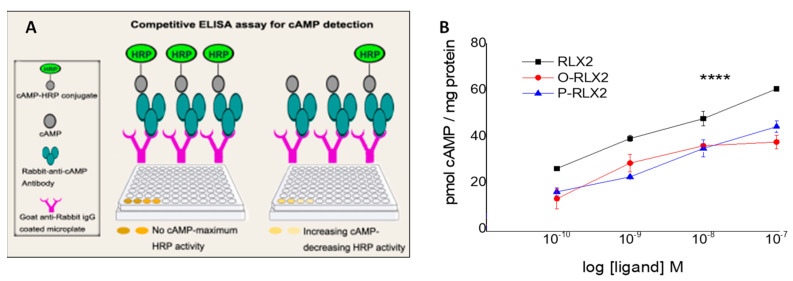
(**A**). Cyclic adenosine monophosphate (cAMP) immunoassay method diagram. (**B**). cAMP dose-response curve after treatment of cells with free RLX2, O-RLX2, or P-RLX2. cAMP concentration was measured with the cAMP immunoassay, and expressed as pmol cAMP per ug of protein, for each dose. **** *p* < 0.0001.

The bioactivity of the three peptide types in solution was studied initially, in order to understand if the bioactivity was also influenced by the applied chemical modifications, as demonstrated by the O-RLX2 stability study (Figure 4), compared to that of RLX2 (Figure 3B). The cAMP immunoassay method, which was applied for measuring the amount of cAMP produced due to incubation of cells with the bioactive peptides, is explained in Figure 5A. As observed in the results reported in Figure 5B, the RLX2 bioactivity was significantly higher compared to the two other peptide forms evaluated, the O-RLX2and the P-RLX2 conjugate. At all peptide concentrations evaluated, RLX2 resulted in the production of approx. double the amount of cAMP (per ug of protein), compared to the other two peptide types (O-RLX2 and P-RLX2). These results suggest that RLX2 peptide oxidation, as well as its conjugation with Palm-L-Glu-OtBu, both resulted in a similar decrease of its (in vitro) biological activity, since P-RLX2 and O-RLX2 were observed to have the same ability to produce cAMP at all the concentrations evaluated herein.

#### 3.5.2. Preservation of Biological Activity by Liposomal Encapsulation

**Figure 6:**
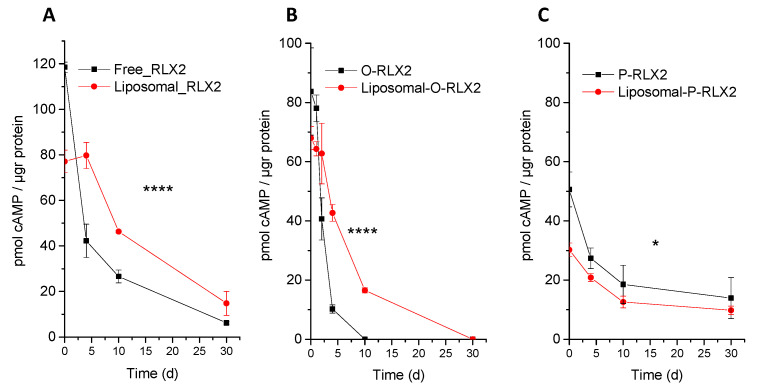
Bioactivity or cyclic adenosine monophosphate (cAMP) production, after treatment of cells with free and liposomal RLX2 peptides that were incubated for various durations in FBS (80% *v*/*v*) at 37 °C; (**A**) RLX2 peptides, (**B**) O-RLX2 peptides, and (**C**) P-RLX2 peptides. **** *p* < 0.0001; * *p* = 0.0176.

Another important question is whether liposome encapsulation could also preserve the bioactivity of the peptides, as demonstrated for their stability (Figure 3 and Figure 4). In Figure 6, the bioactivity of free and the corresponding liposomal peptides (expressed as cAMP production) is measured after incubation of the peptides in FBS for various time periods. The cAMP amounts measured for each peptide cannot be directly compared with the values presented in Figure 5, since the peptides were incubated in FBS before being added to the cells; therefore, in this case, cAMP production was measured in the presence of FBS, while in the dose response experiment, no serum was present. As seen in Figure 6A, RLX2 peptide encapsulation in liposomes resulted in an initial decrease of the peptide bioactivity at time 0, probably due to the fact that liposome-entrapped peptides could not immediately stimulate cAMP production by the cells. A reduced activity, due to reduced substrate permeation through the lipid membrane, was also previously reported for acetylcholinesterase [20]. However, after 3 d of incubation at 37 °C, although the free RLX2 peptides demonstrated a dramatic decrease of their bioactivity (which was decreased to 1/3 of its initial value), the bioactivity of the liposomal RLX2 peptides was fully preserved, indicating a strong preservation of peptide bioactivity, due to peptide encapsulation in liposomes. Similar activity preservation was also observed after 10 d and 30 d of peptide incubation in FBS at 37 °C, for RLX2 peptides. In fact, the half-life of RLX2 bioactivity (duration of incubation at which the peptide lost the 50% of its initial bioactivity) increased from 3.5 d to 9.4 d with liposome entrapment.

Similar results were also observed for the preservation of O-RLX2 bioactivity (Figure 6B), although the bioactivity of this peptide type was initially lower compared to that of native RLX2 (in good agreement with the results of Figure 5), and also decreased faster (compared to RLX2) in both forms evaluated (free [solution] and liposomal) and for all time periods evaluated. Indeed, after 3 d of incubation, the bioactivity of free O-RLX2 peptide was already reduced to the 1/8 of its initial value, while no bioactivity was measured after 10 d of incubation. Again, liposome encapsulation provided significant (*p* < 0.0001) preservation of the bioactivity of O-RLX2 peptide, as also observed for RLX2 (Figure 6B). In fact, the half-life of O-RLX2 bioactivity was increased from 1.65 d to 3.35 d by liposome entrapment.

Concerning the bioactivity of the P-RLX2 conjugate (Figure 6C), the initial cAMP amount value measured at time 0 was even lower compared to the other two peptide types (RLX2 and O-RLX2) when compared to the values measured in the dose–response experiment (Figure 5). In other words, although O-RLX2 and P-RLX2 had similar bioactivities in the dose-response studies (Figure 5), this was not the case in the bioactivity preservation study, where the initial value measured for O-RLX2 was 84.1 ± 2.1, and the value for P-RLX2 was 50.7 ± 3.5 (Figure 6). The latter observation is most possibly due to the presence of serum proteins in the current samples. Perhaps the presence of proteins has a different effect on the conformation of the two peptide types, affecting their cAMP production activity differently. One possibility is that the Palm-L-Glu-OtBu lipid modifier may bind to hydrophobic binding sites of serum proteins to a higher extent compared to the oxidized peptide (that is, less hydrophobic and no lipid modifier is present), being thus partially inactivated (concerning their cAMP production activity). Opposed to the other peptide types, the encapsulation (or better incorporation) of the P-RLX2 peptide in liposomes did not confer any prolongation or enhancement on the peptide bioactivity, but on the contrary resulted in a slightly decreased bioactivity, probably due to reduced substrate permeation through the lipid membrane [20].

## 4. Discussion

Liposome encapsulation and fatty acid conjugation were investigated as methods to retain the stability and prolong the biological activity of RLX2 peptides. To the best of our knowledge this is the first report that has compared the effect of chemical modification and liposome encapsulation of any peptide on the peptide activity. A palmitoyl conjugate of RLX2 peptide (P-RLX2) was synthesized, using *N*-Palmitoyl-l-glutamic acid α-tert-butyl ester γ-succinimidyl ester (Palmitoyl-l-Glu(OSu)-OtBu) as the fatty acid modifier, after identifying an efficient synthetic pathway and the appropriate conditions that conferred a high production yield of the monopalmitate RLX2 peptide conjugate, in the final stage of its synthesis. This peptide modifier is known to delay peptide absorption and extend the half-life of Liraglutide, while renal clearance is also reduced, due to the shielding effect of the fatty acid moiety [21].

RLX2 peptide encapsulating nanosized liposomes were additionally formulated (with mean diameters between 114 nm–160 nm) for all the peptide types, by appropriate methods that confer a high encapsulation. In more detail, the DRV method [22,23], a mild method, which protects the integrity of peptide/protein drugs and confers high encapsulation for aqueous soluble materials, was used for encapsulation of RLX2 and O-RLX2 peptides, and it was found that inclusion of a negatively charged lipid (PG) conferred the maximum encapsulation of these peptides (EE up to 22%), due to the fact that they were oppositely charged at pH 7.40. Protein encapsulation in liposomes has been reported to be dependent on the extent of interactions that occur between the encapsulated proteins and the phospholipid bilayer [34]. For the P-RLX2 lipid conjugate of RLX2 incorporation in the liposome membrane, the thin film method was applied, and it was fully incorporated in the liposomes.

For the reduction of the size of both types of peptide-loaded liposomes, DRVs and MLVs, extrusion was applied, since it was previously reported that extrusion resulted in a much higher retention of heparin in nanosized DRV liposomes compared to sonication [35]. We anticipate that extrusion may additionally be less harmful to such bioactive peptides than sonication; however, this remains to be proven.

It is well known that PEGylated liposomes have a higher integrity compared to non-PEGylated ones (in presence of serum proteins, FBS) [24,30,31], and thereby it was essential to study PEGylated liposomes. In order to coat the RLX2-peptide entrapping liposomes with PEG, a post-PEGylation method was required, since when PEG was added initially (during lipid film formation (conventional method)), a very low encapsulation was realized (2.85%). In good agreement with previous findings, when the stability of RLX2 peptides was evaluated in buffer, the entrapment of the peptide in PEGylated liposomes was demonstrated to confer maximum peptide integrity. However, the peptide integrity in the presence of serum was not affected by liposome PEGylation, at least under the conditions applied herein. In fact the integrity of RLX2 peptide was significantly higher in serum, compared to that measured in buffer (without proteins), probably due to the increased molecular shielding of the peptides by the proteins.

The results of the peptide stability studies indicated that both peptide types, RLX2 (Figure 3) and O-RLX2 (Figure 4) were highly protected against degradation when they were entrapped in liposomes.

The current results about the liposomal forms of RLX2 peptide types cannot be compared with others, since the liposomal encapsulation of RLX2 had not been previously studied as a method to prolong this peptide’s activity; the beneficial effect of a special type of targeted RLX2 liposomes has been reported [4]; however, the properties and/or stability of RLX2 in those formulations have not been reported.

On the other hand, several chemical modifications were previously reported for RLX2. Dicarba RLX2 analogues were found to retain their activity but lose their stability in serum [13], while other synthetic covalently linked dimeric forms of human RLX2 were seen to retain their (receptor binding) activity and also demonstrated improved in vitro serum stability [15]. Nevertheless, a direct comparison of the reported results of RLX2 stability in serum with the current results could not be made, since much lower stabilities in serum were reported by others for the monomeric amidated form of H2 relaxin, and also for native H2 relaxin [13,36], probably due to the fact that different peptide concentrations and a different type of serum (human serum) were used in those studies. In any case, it should be pointed out that, according to previous studies, the in vivo half-life of peptides is always much lower compared to in vitro results, since renal clearance is a major factor that determines the in vivo half-life of peptides [15].

Comparing the retention of the bioactivity of the three peptide types studied herein, from the dose response bioactivity experiments carried out in the absence of proteins, it was indicated that peptide oxidation on Met25 has an activity reducing effect, and palmitoylation of RLX2 with a Palmitoyl-l-Glu-OtBu modifier at the a-amino group of the *N*-terminal amino acid of the Β-chain, had a similar effect on the bioactivity of the peptide as that caused by oxidation (Figure 5). However, when the RLX2 bioactivity was measured in presence of proteins (Figure 6), the results suggested that interactions of the various peptide types with serum proteins affected their bioactivity in different ways, since P-RLX2 had a significantly lower bioactivity compared to O-RLX2, and both latter peptide types had a lower bioactivity compared to RLX2.

Interestingly, although the bioactivity measured for P-RLX2 conjugate was significantly lower than that of RLX2 (and O-RLX2), it was highly preserved. Indeed, after 30 d incubation in FBS at 37 °C, the bioactivity of the conjugate was much higher (approx. 30% of its initial value) compared to the preservation of the bioactivity of the liposomal form of RLX2 peptide (the only other peptide type that retained a significant bioactivity value after 30 d, which was approx. 13% of its initial value). However, due to the higher initial bioactivity of liposomal RLX2, its bioactivity was practically the same as that of P-RLX2 at the 30 d time point. Nevertheless, liposomal RLX2 demonstrated a much higher bioactivity at all other time points evaluated; therefore, according to the current results, entrapment of RLX2 peptide in PEGylated liposomes was the most successful method, among those evaluated herein, for attaining a high and prolonged biological activity of RLX2. Another very interesting finding from the current study is that the stability study resulted for for the free and liposomal forms of RLX2 and O-RLX2 peptides are in good agreement with the results of the bioactivity preservation studies, indicating that such studies may provide good predictions for the preservation of their biological activity.

## 5. Conclusions

The current study proved that encapsulation of RLX2 in PEGylated liposomes could be used as a method to preserve its biological activity. The synthesis of a Palm-L-Glu-OtBu conjugate of RLX2, where this peptide modifier was linked at the a-amino group of the *N*-terminal amino acid of the Β-chain of RLX2, resulted in a significant loss of its biological activity, although the remaining activity was highly preserved for up to 30 d, under the conditions applied in the current study.

## Figures and Tables

**Table 1:** RLX2 liposome physicochemical properties. Liposomes were prepared by the DRV method, and each value reported is the mean of at least three different samples; corresponding SD values are reported.

Lipid Composition	EE (%)	Drug/Lipid (mol/mol × 10^4^)	z-Average Size (nm)	PDI	ζ-Potential (mV)
PC/Chol (2:1)	4.03 ± 0.47	1.28 ± 0.14	113.8 ± 1.4	0.093 ± 0.012	−4.95 ± 0.21
PC/PG/Chol (8:2:5)	22.0 ± 2.1	7.08 ± 0.67	132.4 ± 1.9	0.075 ± 0.010	−26.9 ± 1.3
PC/PG/Chol/PEG(8:2:5:1.3)	2.85 ± 0.63	0.91 ± 0.28	128.8 ± 1.8	0.098 ± 0.015	−8.3 ± 1.8
PC/PG/Chol/PEG(8:2:5:1.3) (Post-Pegylation) 1 h	16.9 ± 2.9	5.32 ± 0.86	153.3 ± 4.8	0.194 ± 0.021	−11.6 ± 1.5
PC/PG/Chol/PEG(8:2:5:1.3) (Post-Pegylation) 2 h	17.4 ± 1.1	5.49 ± 0.33	158.7 ± 4.5	0.198 ± 0.019	−8.61 ± 0.95

**Table 2:** Physicochemical properties of O-RLX2 (O) loaded and P-RLX2 (P) incorporating liposomes (PC/PG/Chol (8:2:5) or PC/PG/Chol/PEG (8:2:5:1.3)). Each value reported is the mean of at least three different samples; corresponding SD values are reported.

Peptide-Lipid Composition	EE (%)	Drug/Lipid (mol/mol × 10^4^)	z-Average Size (nm)	PDI	ζ-Potential (mV)
O-PC/PG/Chol	22.6 ± 2.6	7.22 ± 0.81	138.5 ± 2.2	0.086 ± 0.010	−22.1 ± 1.3
O-PC/PG/Chol/PEG	21.0 ± 2.5	6.40 ± 0.79	153.8 ± 2.8	0.198 ± 0.015	−11.7 ± 3.1
P-PC/PG/Chol/PEG	~100	~30	146.2 ± 5.1	0.202 ± 0.015	−11.3 ± 1.04

## Data Availability

The data presented in this study are available on request from the corresponding author.

## Supplementary Materials

The following supporting information can be downloaded at https://www.mdpi.com/article/10.3390/biom12101362/s1, Figure S1: Standard curve of RLX2 in PBS solution; Figure S2: Standard curve of RLX2 in PBS (A) or 80% FBS (B); Figure S3: Standard curve of O-RLX2 in PBS solution; Figure S4: Standard curve of O-RLX2 in PBS (A) or 80% FBS (B); Figure S5: Representative graphs of liposome size distribution;
