# Supplementary material for: Liposomal Entrapment or Chemical Modification of Relaxin2 for Prolongation of Its Stability and Biological Activity
Source: Biomolecules. 2022 Sep 24;12(10):1362. doi: 10.3390/biom12101362 (PMC9599704; doi:10.3390/biom12101362)
Supplement: Supplementary file 1 [file biomolecules-12-01362-s001.zip › Figure S1-R1.pdf]

Supplementary Figure S1 for Paper :" Liposomal entrapment or chemical modification of Relaxin2 for prolongation of its stability and biological activity "

By: George Kogkos , Foteini Gkartziou, Spyridon Mourtas , Kostas K. Barlos, Pavlos Kletetsanis, Kleomenis Barlos, and Sophia G. Antimisiaris*

A.

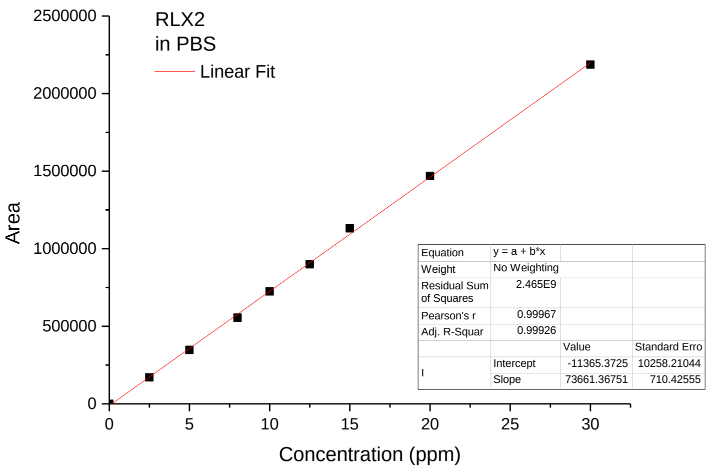

B.

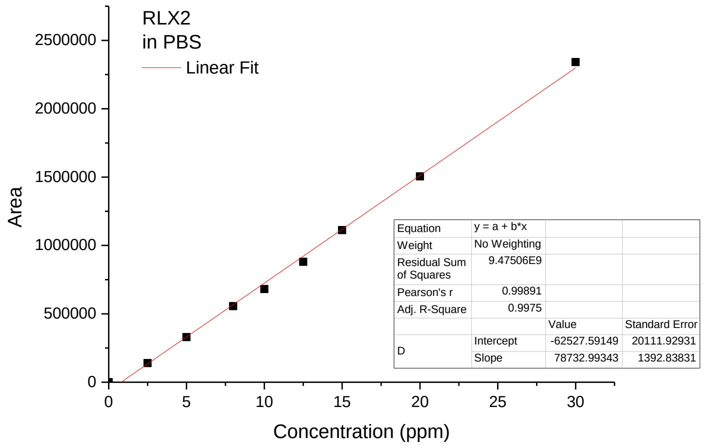

B.

Figure S1: Standard curve of RLX2 in PBS solution, alone (A) or in presence of 1 mg/ml lipid (PC:PG:Chol) (B)
